# A Puzzling Mast Cell Trilogy: Anaphylaxis, MCAS, and Mastocytosis

**DOI:** 10.3390/diagnostics13213307

**Published:** 2023-10-25

**Authors:** Theo Gülen

**Affiliations:** 1 Department of Respiratory Medicine and Allergy, Karolinska University Hospital Huddinge, 141 86 Stockholm, Sweden; theo.gulen@regionstockholm.se; 2 Department of Medicine Solna, Division of Immunology and Allergy, Karolinska Institutet, 171 77 Stockholm, Sweden; 3 Department of Medicine Huddinge, Karolinska Institutet, 141 52 Stockholm, Sweden; 4 Mastocytosis Centre Karolinska, Karolinska University Hospital Huddinge, 141 86 Stockholm, Sweden

**Keywords:** anaphylaxis, MCAS, mastocytosis, *KIT* D816V, tryptase, mediator-release symptoms, hereditary alpha-tryptasemia, atopy

## Abstract

Our knowledge of biology and mast cell (MC) function, as well as disorders associated with the pathologic activation of MCs, has evolved over the last few decades. Anaphylaxis, mast cell activation syndrome (MCAS), and mastocytosis are interrelated yet distinct conditions within the spectrum of mast cell activation disorders. Nevertheless, all three conditions can co-exist in one and the same patient, as pathologic MC activation is the key finding in all three. When mediator release is excessive and involves multiple systems, anaphylaxis and MCAS may occur. Furthermore, mastocytosis is a clonal disorder of MCs and often presents with anaphylaxis and MCAS. Nevertheless, in some cases, even the proliferative and accumulative features of MCs in mastocytosis can account for symptoms and disease progression. In each case, diagnosis can be only made when the diagnostic consensus criteria are fulfilled. The current article aims to provide a concise clinical update and pinpoint the main difficulties in diagnosing these puzzling disorders of MCs in medical practice.

## 1. Introduction

Mast cells (MCs), as multifunctional immune cells, orchestrate the typical allergic conditions wherein the activation of these cells by allergens, including pollen, food, medication, and the venom of stinging insects, leads to the degranulation and elaboration of the inflammatory mediators responsible for regulating the acute dramatic inflammatory response [1,2,3,4,5]. Nevertheless, MCs also participate in many physiologic processes, including maintaining homeostasis, tissue inflammation and repair, innate and adaptive immune responses, immune tolerance, and host defense [6,7,8].

MC activation may be seen in a variety of clinical contexts and pathologies. Historically, the clinical symptoms arising from MC activation have primarily been studied in hypersensitivity disorders, such as IgE-dependent allergic inflammation or other immunologic reactions; however, more recently, they have also been analyzed in the context of clonal MC disorders, such as mastocytosis [9,10,11,12]. Symptoms related to MC activation may be local or systemic and acute or chronic, and may range from mild to severe. Acute MC activation is often seen in allergic reactions and may be associated with the symptoms and signs of anaphylaxis [12,13]. In severe reactions, MC activation can be documented due to a substantial increase in serum tryptase levels [14]. In such patients, an MC activation syndrome (MCAS) may be diagnosed when the presence of the MCAS criteria is confirmed [15,16,17]. Moreover, mastocytosis may be diagnosed when clonally aberrant MCs accumulate in one or more organs, including the skin, bone marrow, and intestine [11,18]. In general, severe or even life-threatening MC activation events usually occur in patients with mastocytosis, as clonally aberrant MCs induce a “hyperreactive” state in MCs and/or decreased MC activation thresholds resulting in the hyper-releasability of MCs [12,19]. 

The present article aims to discuss the diagnostic and clinical features of anaphylaxis, MCAS, and mastocytosis, as well as the state of dysfunctional MCs in light of recent developments, to help practicing physicians, including allergists, immunologists, dermatologists, and hematologists, navigate these diagnostically challenging clinical scenarios. 

## 2. Mast Cells and Mast Cell Activation

MCs are granulated cells that have a widespread distribution in all vascularized tissues [1,2,3,4,5]. The multifunctional capacity, heterogeneity, and plasticity of MCs enable them directly or indirectly to regulate innate and adaptive immune responses by communicating with other cells in the immune system [4,5,6]. The local microenvironment directly affects their maturation, phenotype, function, and ability to respond to internal or external stimuli by releasing biologically active mediators [4,5].

**Figure 1:**
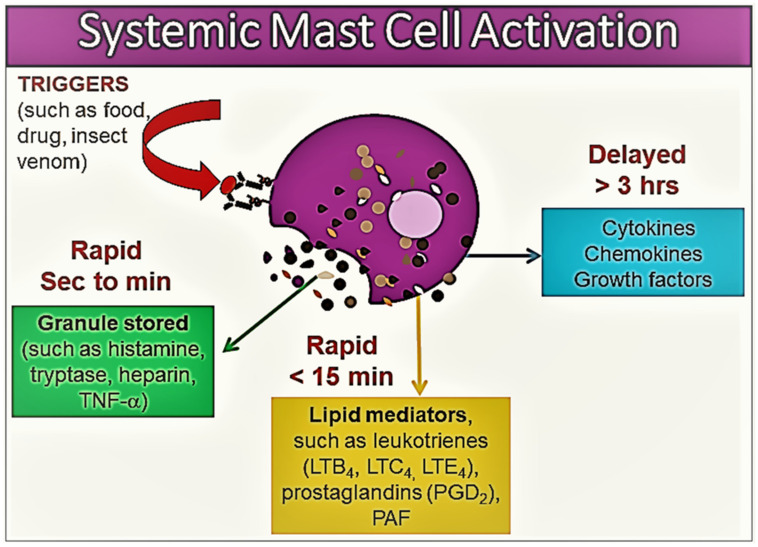
An illustration of the release of various mast cell mediators in the context of systemic mast cell activation. The mast cell inflammatory mediator profile shows heterogeneity according to the tissue microenvironment, the severity of mast cell activation, and the release of mast cell products. Please see the related text for further discussion.

MCs express numerous surface receptors and are involved in the initiation and perpetuation of allergic inflammation. MCs can be activated by various mechanisms, most often through the cross-linking of immunoglobulin E (IgE) molecules bound to their surface by high-affinity FcεRI receptors [3,8,9]. Non-IgE-mediated mechanisms leading to MC activation include activation pathways through toll-like receptors, stem cell factor receptors (KIT = CD117), complement receptors C3a and C5a, and surface G protein-coupled receptors, including MRGPRX2 [9,20]. When activated, in a sequential order, MCs release various biologically active mediators [1,8,9] (Figure 1).

Within minutes of activation, MC degranulation leads to the release of the preformed mediators stored in the MC granules, including histamine, serotonin, heparin, chondroitin sulphate, tryptase, chymase, carboxypeptidase, and TNF-α [1,8,9,10]. This first stage is followed by the de novo synthesis of membrane lipid-derived mediators, particularly prostaglandin (PG) D2, cysteinyl leukotrienes (LTC4, D4, and E4), and platelet activating factor (PAF) [8,9,10]. At the next stage, MC activation results in the synthesis of a variety of pro- and anti-inflammatory cytokines, including TNF-α, GM-CSF, IL-1, IL-3, IL-4, IL-5, IL-6, IL-13, IL-1RA, chemokines (such as IL-8, CCL-2, CC-3, CCL-5, and CXCL-8), growth factors (such as transforming growth factor-beta 1 (TGF-β1), stem cell factor (SCF), fibroblast growth factor (FGF), nerve growth factor (NGF), platelet-derived growth factor (PDGF), and vascular endothelial growth factor (VEGF)), and interferons [1,8,9,10] (Figure 1).

The roles of mediators differ in the clinical manifestations of MC disorders, and related symptoms may show great heterogeneity according to the tissue in which MC activation occurs, as well as the trigger that causes the MC activation [10]. MC-derived mediators, such as histamine, leukotrienes, prostanoids, and PAF, regulate vascular instability and the barrier dysfunction of endothelial cells, and contribute to oedema formation, hypovolemia, and shock [10,21,22]. Histamine, specifically, can contribute to many classic manifestations associated with acute allergic reactions such as pruritus, flushing, nausea, gastric hypersecretion, nasal congestion, and wheezing. Histamine has been shown to bind to four receptors: H1 (responsible for airway and mucosal inflammation, attention, sleep–wake cycle regulation, and food or water intake), H2 (involved in the relaxation of the airway and blood vessel smooth muscle and in gastric acid secretion), H3 (involved in neuroregulation and mediator release modifying cognition, sleep–wake cycle regulation, and inflammation), and H4 (involved in the modulatory effects on inflammatory responses) [10]. Furthermore, histamine, kinins, leukotrienes, and PAF may be responsible for the opening of endothelial gap junctions, resulting in angioedema, pulmonary edema, and gastrointestinal symptoms (such as crampy abdominal pain and diarrhea related to mucosal swelling). Histamine, PGD2, leukotrienes, PAF, and prostanoids are responsible for wheezing and mucus hypersecretion [10,21,22]. Cytokines, chemokines, leukotrienes, and histamine may lead to neurological symptoms including headaches, fatigue, a sense of impending doom, and confusion [10,21,22]. Studies suggest that the activation of the contact system and the secretion of the plasminogen activator and heparin may lead to coagulation abnormalities and bleeding. Likewise, blood basophils may also participate in allergies through FcεRI-dependent reactions by releasing a similar profile of mediators [1]. However, not all hypersensitivity reactions may involve basophils, even if the reaction is systemic [1]. Moreover, some of the relevant mediators and repair molecules are produced and released exclusively by MCs but not by basophils [1].

## 3. Disorders Associated with Mast Cell Activation and Nomenclature

**Figure 2:**
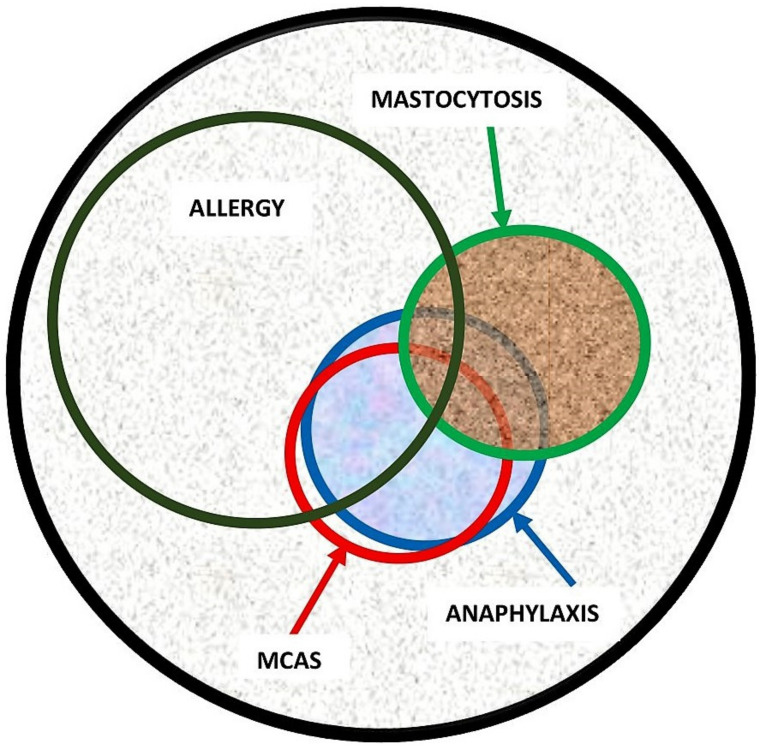
Illustration of disorders related to pathologic mast cell activation and association between allergy, anaphylaxis, mast cell activation syndrome (MCAS), and mastocytosis. While there is a significant overlap between the conditions, the size of the circle does not accurately represent the true percentage of overlapping. For example, the estimated overlap between anaphylaxis and mastocytosis is approximately 35%. However, there is currently a lack of systematic studies providing a precise estimation of the overlap between anaphylaxis and MCAS.

Evidence in the recent literature suggests that the spectrum of disorders related to mast cell activation is broad and includes IgE-dependent allergic inflammation and other immunologic and inflammatory reactions. Activated MCs not only, however, participate in the pathogenesis of hypersensitivity disorders but are also involved in an emerging group of conditions, so-called mast cell activation disorder (MCAD), such as mastocytosis [12,23]. Pathologic MC activation is a key finding in both hypersensitivity and MCAD, albeit caused by entirely different mechanisms. Therefore, patients with both disorders present with overlapping symptomatology due to inappropriate MC mediator release (Figure 2). Notably, both exogenously triggered allergies and endogenously triggered MCAD may cause anaphylaxis, which can be described as a “unique” condition representing a common clinical feature of these two distinct conditions [12].

The signs and symptoms of disorders associated with MC activation and mediator release may range from tissue-specific events such as localized itching or nasal congestion to more systemic symptoms that result from widespread MC activation. Examples of the tissue-specific consequences of MC activation include urticaria, allergic rhinitis, or asthma, and the symptoms, in most instances, are limited to the area of interaction with the trigger, although generalized-tissue-specific symptoms also are possible, such as in the case of chronic idiopathic urticaria. Systemic MC activation reactions, such as anaphylaxis and MCAS, present with symptoms including two or more organ systems (skin: urticaria, angioedema, and flushing; gastrointestinal: nausea, vomiting, diarrhea, and abdominal cramping; cardiovascular: hypotensive syncope or near-syncope and tachycardia; upper and lower respiratory: conjunctival injection, nasal pruritus, nasal stuffiness, rhinorrhea, dyspnoea, and wheezing).

It is worth mentioning the nomenclature once again here. Experts often do not speak the same language, as they either use different terms for the same concepts, or the same term for different concepts, probably without being aware of it. For instance, the terms “mast cell activation disorder” (MCAD) and “mast cell activation syndrome” (MCAS) are often used interchangeably in the literature. Although they can refer to similar conditions involving abnormal MC activation, there are certain differences in their usage and scope. Although MCAD is a broader term encompassing a range of conditions characterized by pathologic MC activation, MCAS is a specific type of MCAD that is characterized by severe, recurrent episodes of systemic MC activation [12,23]. MCAS typically involves the release of excessive mast cell mediators throughout the body, resulting in a wide range of symptoms affecting multiple organ systems as mentioned in detail below in the MCAS section of this paper.

### 3.1. Mastocytosis

Mastocytosis refers to a complex heterogeneous multisystem disorder characterized by a pathologic activation and accumulation of clonally aberrant MCs in one or more organs, including the skin, bone marrow, liver, spleen, lymph nodes, and gastrointestinal tract [11,18]. The existing evidence suggests that it is a rare condition, and in recent studies, its prevalence is estimated to be 1 in 10,000 persons [24,25,26,27]. In general, mastocytosis can be divided into cutaneous mastocytosis (CM), where only the skin is affected, and systemic mastocytosis (SM), involving at least one additional organ other than the skin [11,18]. CM is the main form of the disease in children and patients are generally observed to have the onset of the disease within the first year of life. The most common form of skin involvement is maculopapular cutaneous lesions, also known as urticaria pigmentosa (UP) [11,18]. Pathognomonic Darier’s sign is a key feature and criterion of cutaneous lesions in mastocytosis; it is defined by swelling and redness after stroking or rubbing of the lesional skin in an affected individual. The sensitivity of Darier’s sign is over 90%. However, a skin biopsy should be obtained in doubtful cases and increased numbers of MCs in the upper dermis with a predilection to perivascular areas can confirm diagnosis.

Mastocytosis in children is most often limited to the skin and the prognosis is good, as skin lesions are resolved in most patients by adolescence. By contrast, patients with adult-onset mastocytosis have a persistent disease and may or may not present with skin lesions. In the majority of adult patients with maculopapular lesions (most frequently monomorphic, i.e., UP), MC infiltrates are also found in the bone marrow, corresponding to the final diagnosis of SM [11,18]. SM is diagnosed according to the World Health Organization (WHO) criteria, which consist of one major and four minor criteria, and the diagnosis requires demonstration of the major criterion (multifocal aggregates of MCs) along with at least one minor criterion, or three minor criteria in extracutaneous, most often bone marrow, biopsy materials (Table 1) [11,18]. Otherwise, adult patients with the typical skin lesions of mastocytosis are denoted as the mastocytosis in the skin (MIS) [18]. This is a provisional entity that is reserved for adult cases with cutaneous involvement (typically monomorphic maculopapular cutaneous lesions, i.e., UP), but in whom SM has not been ruled out yet. Although rare, there are also adult patients with skin lesions who do not show systemic involvement. In adult patients with true CM, the criteria for SM are not fulfilled, even if clonal MCs can be detected in the extracutaneous organs [18].

The most prevalent form of SM in adults is indolent SM (ISM) [26,27]. These patients have a normal life expectancy compared to the age-matched general population [26,27]; however, they may experience a variety of unpleasant symptoms. For instance, increased susceptibility to the development of malign melanoma has been reported in ISM patients [28]. In addition, advanced variants of SM, including aggressive SM (ASM), SM with associated hematologic neoplasm (SM-AHN), and MC leukemia (MCL), can occur in rare cases [18]. These patients generally have a large burden of clonally aberrant MCs in their bone marrow and carry a poor prognosis [18].

SM is a complex disease with diverse clinical manifestations ranging from asymptomatic disease to a highly aggressive course with multisystem involvement [11]. Most patients with ISM have symptoms caused by the inappropriate release of MC mediators, such as histamine, proteases (e.g., tryptase, chymase, and carboxypeptidase), and lipid-derived mediators (e.g., cysteinyl leukotrienes and prostaglandin D2) [11]. Symptoms may be acute or chronic and result from the local or remote effects of these mediators, which may act on multiple organ systems to induce so-called MC mediator release symptoms. Patients may present with diverse clinical findings, including flushing, pruritus, palpitations, dizziness, hypotension, syncope, breathing difficulties, abdominal pain, nausea, vomiting, diarrhea, headache, sweating, lethargy, fatigue, impaired concentration, irritability, anxiety, depression, arthralgia, myalgia, and osteoporosis [11]. However, not all patients experience all these manifestations; therefore, this heterogeneity is still unexplained. The mediator levels do not usually show a clear association with the clinical phenotypes, although the baseline levels of mediators including tryptase, histamine, and prostaglandin D2 are generally elevated [29]. Nevertheless, a history of flushing is a cardinal symptom [30]. In addition, some subjects may experience isolated symptoms, whereas others develop a constellation of signs and symptoms indistinguishable from that of anaphylaxis, which can be life-threatening [19,30]. Typically, patients suddenly feel very warm and then experience palpitations, dizziness, and a decrease in blood pressure due to systemic vasodilatation, which often leads to syncope [30]. Acute attacks may be brief or prolonged, but the duration is usually 15 to 30 min [30]. Patients often experience severe fatigue lasting around 24 h following spells [30]. Although specific triggers causing MC mediator release symptoms vary greatly among patients, exogenous and endogenous triggers may include physical exertion, cold, heat, insect venoms, the consumption of alcohol, infections, nonsteroidal anti-inflammatory drugs (NSAIDs), and emotional stress [11].

Advanced SM patients may also have symptoms of MC mediator release; interestingly, however, the occurrence of anaphylaxis due to the excessive release of MC mediators is less common compared with that in patients with ISM. These patients mainly experience symptoms due to MC infiltration and uncontrolled accumulation, including cytopenia, hepatosplenomegaly, lymph adenopathy, liver dysfunction, ascites, osteolytic bone lesions, and pathologic weight loss [11].

**Table 1:** Diagnostic criteria of systemic mastocytosis (SM) and monoclonal mast cell activation syndrome (MMAS) (adapted from references: [11,18,31,32]). Altogether, there is one major criterion and four minor criteria.

**SM**	**Diagnosis is confirmed if patient expresses one major criterion and one minor criterion or expresses three minor criteria in extracutaneous organ biopsy specimens**
**Major criterion** **Minor criteria**	Multifocal aggregates of MCs (≥15 MCs per cluster) in biopsy sections In MC infiltrates in extracutaneous biopsy sections, >25% of the MCs (CD117+) are spindle-shaped or have atypical morphology Presence of an activating KIT mutation at codon 816, generally D816V, in bone marrow, blood, or other extracutaneous organ(s) Detection of aberrant MC clones expressing CD117 with CD25 and/or CD2 and/or CD30 in bone marrow or blood or another extracutaneous organ(s) Baseline serum tryptase persistently exceeds ≥20 ng/mL
**MMAS**	**Diagnosis requires presence of one or two minor criteria of SM**
	Presence of an activating KIT mutation D816V, in bone marrow, blood or other extracutaneous organ(s) AND/OR 2. Detection of aberrant MC clones expressing CD117 with CD25 and/or CD2 and/or CD30 in bone marrow or blood or another extracutaneous organ(s)

### 3.2. Monoclonal Mast Cell Activation Syndrome

Monoclonal mast cell activation syndrome (MMAS) is a recently introduced variant of clonal MC disorders [31,32] and most such patients experience severe anaphylaxis episodes presenting with profound cardiovascular manifestations such as hypotension and syncope in the absence of urticaria. Although patients with MMAS have detectable clonal MCs expressing the D816V mutation and/or CD25+ aberrant markers, they do not fulfill the WHO criteria for SM diagnosis [11,18]. In addition, MMAS patients lack the typical skin changes in mastocytosis (Table 1). Furthermore, these patients have a normal to low burden of MCs and their serum baseline tryptase (sBT) levels generally range from 10 to 20 ng/mL but may also be within normal ranges.

The diagnosis of MMAS requires a high degree of clinical suspicion and confirmation by means of bone marrow biopsy. A diagnosis should be considered in patients presenting with symptoms of hypotensive anaphylaxis. Peripheral blood *KIT* D816V mutational analysis with an allele-specific PCR method can be used as a screening tool in these patients; however, the absence of this mutation in the peripheral blood does not rule out MMAS. Hence, a bone marrow biopsy should be considered in highly suspected cases.

This condition continues to exhibit limited understanding in terms of its natural course and prognosis and the development of comorbidities such as osteoporosis. Although some of these patients may be found to have SM in future biopsies, this is most probably because of misdiagnosis in the initial biopsies. According to personal observations, most MMAS patients do not progress into SM. Moreover, the spontaneous resolution of MMAS has not been described to date.

Another relevant point to highlight here is that MMAS and MCAS are related but distinct terms. These somewhat incongruous entities may complicate the nomenclature further. However, in certain patients with MMAS, MCAS may coexist when the diagnostic criteria for both conditions are met simultaneously (please see the MCAS criteria below in Section 3.3).

### 3.3. Anaphylaxis

Anaphylaxis is a common medical emergency and a life-threatening acute systemic hypersensitivity reaction that may lead to death by airway obstruction or cardiovascular collapse if not promptly treated. Anaphylaxis is one of the most well-known examples of systemic MC activation disorders and is caused by the abundant release of diverse MC mediators, leading to a constellation of varied symptoms from different organ systems [12,13]. The term anaphylaxis encompasses both IgE-mediated reactions and non-IgE-mediated mechanisms. Although this difference may impact allergen counselling, it has no importance in the acute management of the patient.

The data regarding the incidence and prevalence of anaphylaxis are somewhat inconsistent due to the fact that a consensus regarding its exact definition does not currently exist [33]. Studies from the USA suggest an incidence of up to 40–50 people per 100,000 person-years [34,35], whereas studies from Europe suggest a lower incidence of 1.5–7.9 per 100,000 person-years [36,37]. Moreover, the lifetime prevalence of anaphylaxis has been estimated to be approximately 0.3% [38]. There are studies showing an increase in admissions with anaphylaxis over the last two decades [38]. Although rare, deaths may also occur at a rate of 1 per three million population per year [39]. Foods, drugs, and the venom of stinging insects appear to be the most common elicitors of anaphylaxis, although the prevalence of triggers varies in children and adults. Food-induced anaphylaxis is the most common cause in children corresponding to 80–92% of anaphylaxis [40], whereas Hymenoptera venom and drug-induced anaphylaxis are the dominating elicitors among adults [41]. It should be also noted that although most anaphylactic reactions occur rapidly, delayed reactions, with onset up to 10 h after ingestion, may occur for some food allergens, e.g., with an alpha-Gal-induced red meat allergy [33].

Anaphylaxis concurrently affects multiple organ systems, and its diagnosis may be challenging, as the fine line differentiating an allergic reaction from anaphylaxis is not always easily discernible. Furthermore, the lack of globally recognized diagnostic criteria of anaphylaxis has not only long caused the failure of recognition and delayed treatment in patients but also hampered research facilities. Recently, however, multinational, multidisciplinary symposia were convened to achieve an international consensus on the clinical criteria for the diagnosis of anaphylaxis [42]. Accordingly, the current diagnostic criteria require the concurrent occurrence of symptoms from at least two organ systems that are related to the cutaneous, gastrointestinal, respiratory, and cardiovascular systems. Table 2 illustrates the clinical criteria of anaphylaxis in different contexts. The required organ system involvement varies depending on whether there is a “likely” or “known” trigger for the actual patient. Exceptionally, in the context of a confirmed allergy (e.g., insect venom or drug) for the given patient, an anaphylaxis diagnosis can be made only due to cardiovascular system involvement (hypotension and/or syncope) after re-exposure to the allergen. Additionally, even when there is no likely cause of the reactions, as in unprovoked anaphylaxis, when the onset of illness is acute, a diagnosis of anaphylaxis can be still made when either reduced blood pressure (or associated symptoms, such as syncope) and/or respiratory compromise or laryngeal oedema are present, accompanied by the involvement of skin–mucosal tissue symptoms [42]. The diagnostic criteria have been widely adopted and validated both retrospectively [43] and prospectively [44]. They were found to be 95% sensitive and 71% specific in a prospective validation study among emergency department patients [44]. Hence, it is critical for emergency department providers to consider anaphylaxis in the differential diagnosis for patients whose symptoms overlap with those of anaphylaxis, including upper airway obstruction, acute asthma, angioedema, flushing, hypotension, syncope, as delayed treatment with epinephrine may cause unexpected adverse outcomes, including fatality.

At present, anaphylaxis remains a clinical entity and its understanding for an allergist is still limited with respect to factors determining its severity and underlying intracellular mechanisms. However, existing clinical observations support the notion that anaphylaxis comprises a heterogeneous group of conditions regarding the nature and route of exposure to triggers, organ involvement, severity, and time course [12]. For instance, food-induced anaphylaxis is a leading cause in children, although venom- or drug-induced anaphylaxis account for the majority of adult cases. The distinctions are not only limited to triggers; even the clinical manifestations differ. Mortality is rare in children and occurs mostly in adult patients due to cardiovascular failure. Fatal anaphylaxis has also been associated with a lack of cutaneous symptoms during the anaphylactic episode.

To overcome these obstacles and recognize severe vs. milder reactions, there have been several attempts to develop severity grading systems for acute allergic reactions, including anaphylaxis [45,46,47,48]. However, they have not been widely implemented due to the lack of a uniformly accepted, validated grading system. Thus, there is currently an unmet need for an appropriately developed and validated severity scoring system for acute allergic reactions to harmonize clinical care and facilitate research.

### 3.4. Mast Cell Activation Syndrome

**Figure 3:**
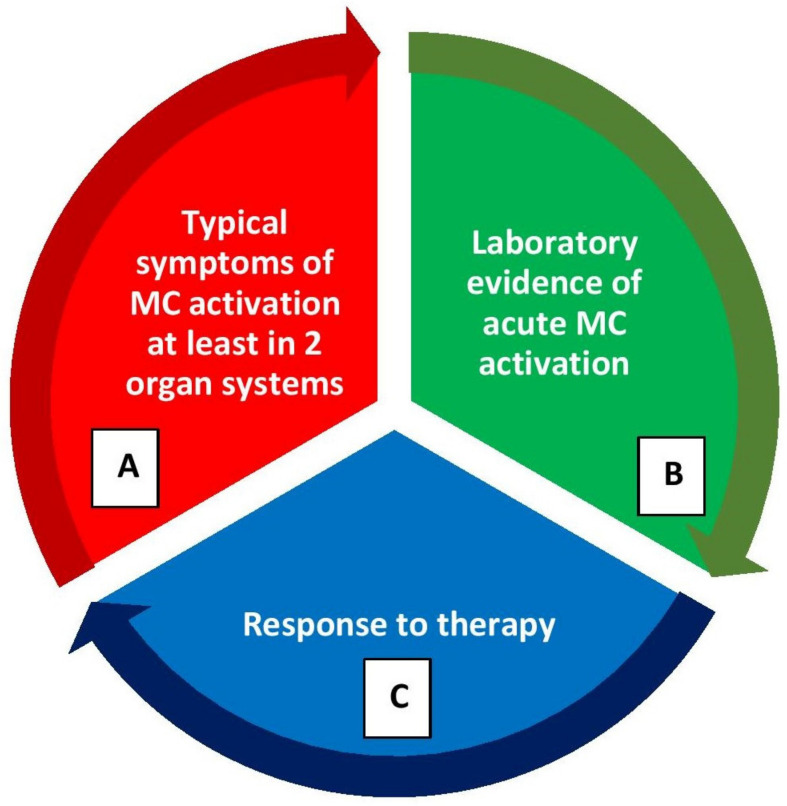
Diagnostic criteria for mast cell activation syndrome (MCAS) (please refer the text for further explanation). (**A**) Clinical criterion, (**B**) laboratory criterion, (**C**) response criterion. All three criterion should be fulfilled to confirm a diagnosis of MCAS. After the diagnosis, patients should be further evaluated for the classification of MCAS.

Mast cell activation syndromes (MCASs) represent a heterogeneous group of disorders that may have clonal or nonclonal etiologies. MCAS may be diagnosed when the symptoms of MC activation are systemic (involving more than one organ system), severe, and recurrent and the MCAS criteria are fulfilled [15,16,17]. There are three sets of criteria required for an MCAS diagnosis as illustrated in Figure 3: (1) the presence of typical, severe, episodic MC activation symptoms in ≥2 organ systems, including cutaneous, cardiovascular, gastrointestinal, and upper/lower respiratory symptoms; (2) the detection of a substantial transient increase in a validated marker of MC activation during the symptomatic event; (3) the control of symptoms with MC mediator-targeting drugs [15,16,17].

The best validated surrogate marker of MC activation is tryptase [14]. The tryptase levels obtained within 4 h of a suspected MC activation episode should be compared with the sBT (i.e., a recent tryptase sample drawn prior to the event or within 48 h after the event) levels. A formula of 1.2 × sBT + 2 ng/mL is used to calculate the minimal increase required to diagnose MC activation [17,49]. For instance, if the sBT level is 15 ng/mL, a level within 4 h of an MCAS episode of 20 ng/mL would be considered significant according to the above-mentioned formula [16,17,49]. Optimally, at least two such elevations should be considered diagnostic following acute, recurrent episodes; however, in practice, this is not always achievable and even one such measurement may be satisfactory.

When tryptase levels are not available, other validated mediators of MCs, such as urinary metabolites of histamine, prostaglandin D2, and leukotriene C4, can be measured in a 24 h or spot collection of the urine specimens collected after the patient empties their bladder during an acute event to confirm MC activation [17]. However, the sensitivity and specificity of these markers, as well as the minimal increases and cut-off levels diagnostic for MC activation, have not yet been established [17]. Recently, it has been suggested that levels greater than 30% above the upper limits of normal can be considered pathologic [50]. Nevertheless, assays are difficult to perform and only available in a few limited laboratories. Moreover, unlike tryptase, the other tests have not been standardized or compared in well-conducted studies.

Finally, MCAS diagnosis also requires a favorable response to agents that act as MC stabilizers or inhibitors of MC mediators. These include histamine receptor antagonists (H1- and H2-antihistamines), leukotriene blockers, MC stabilizers, and aspirin or nonsteroidal anti-inflammatory agents (NSAIDs) [12,51,52]. Selected cases may require immune suppression or cytoreductive therapy as well, especially for patients suffering from aggressive variants of mastocytosis [12,51,52].

Once a diagnosis of MCAS has been confirmed, further classification is necessary. MCAS has been classified into three variants [16,17]. Some patients with MCAS may have concurrently clonal MCs in bone marrow as in mastocytosis (systemic or cutaneous) or MMAS [16,17]. Patients with clonal mast cell disorders generally have varying degrees of expansion of the MC compartment derived from a progenitor with a genetic defect that presumably reduces the cell’s threshold for activation. These patients may have elevated sBT levels, carry *KIT* D816V mutations in lesional MCs, or have other markers of MC clonality, such as aberrant CD25 expression. Such MCAS patients are considered to have primary (i.e., clonal) MCAS and its diagnosis can only be made after an extracutaneous biopsy, most often after a bone marrow biopsy [16,17]. Thus, patients with clonal MCAS are required to fulfill the diagnostic criteria of both MCAS and clonal MC disease.

Nevertheless, the majority of patients with symptoms due to episodic, recurrent MC activation have non-clonal disorders. Thus, secondary MCAS results in symptoms of MC activation through IgE-mediated (such as food-, drug-, or Hymenoptera-venom-induced anaphylaxis) and non-IgE-mediated processes. These patients are cared for by allergists/immunologists. Moreover, in occasional cases, a patient with severe, recurrent MC activation may have an unremarkable work-up for allergic causes and have no evidence of clonal MC disease (usually ruled out after a bone marrow examination). These patients are considered for diagnoses of idiopathic anaphylaxis (IA) or idiopathic (non-clonal) MCAS, depending on the criteria patients fulfill. Furthermore, in some other cases, a primary MC activation (clonal) disorder may coexist with secondary MCAS (e.g., IgE-mediated hypersensitivity/MC reactions to food, medication, or stinging insect venoms). These patients are categorized as having combined or mixed MCAS.

In general, the signs and symptoms of recurrent IgE-mediated anaphylaxis may be the initial presentation of secondary or combined MCAS, whereas IA, e.g., unprovoked anaphylaxis can consist of the initial symptoms of clonal or idiopathic MCAS. However, it is worth mentioning here that not all anaphylaxis episodes fulfill the diagnostic criteria of MCAS, nor do all MCAS episodes reach the severity of anaphylaxis. For instance, some patients with systemic MC activation may have a lesser severity of symptoms that do not meet the definition of anaphylaxis. A typical such scenario is mastocytosis patients with unprovoked flushing episodes associated with abdominal pain. It is more appropriate that such patients are considered for a diagnosis of clonal MCAS rather than IA, as opposed to a patient who experiences hypotensive syncope or respiratory compromise combined with flushing episodes [53].

Although the diagnostic criteria and classification for MCAS have been established by an international (European Union-/US-based) consensus group during the last decade [15,16,17], there is still an ongoing debate about the use of the term MCAS in various groups of patients, and controversies remain. For instance, many patients with suspected MCAS and signs of MC activation do not fulfill the MCAS criteria [17]. In these patients, MC activation may be suspected as the major clinical problem, but it is a significant challenge to prove with certainty that the clinical features and symptoms are indeed derived from MC-dependent reactions and mediator release. Some of these patients may suffer from MC activation disorders (MCADs) or non-specified MC activation reactions, or attributed symptoms may not all be related to MC activation [23]. In these patients, local MC activation, less severe MC activation, or MC activation potentially involving only a limited set of mediators or only one organ system may be implicated, whereas other patients do not have an MCAD. Thus, it should be emphasized here that MCAS cannot be diagnosed in patients presenting with less severe and/or chronic symptoms of MC activation, as only clinically relevant symptoms of severe, systemic, and episodic MC activation can be classified as having MCAS [23].

## 4. Factors Determining the Severity of Mast Cell Activation and Mediator Release

**Figure 4:**
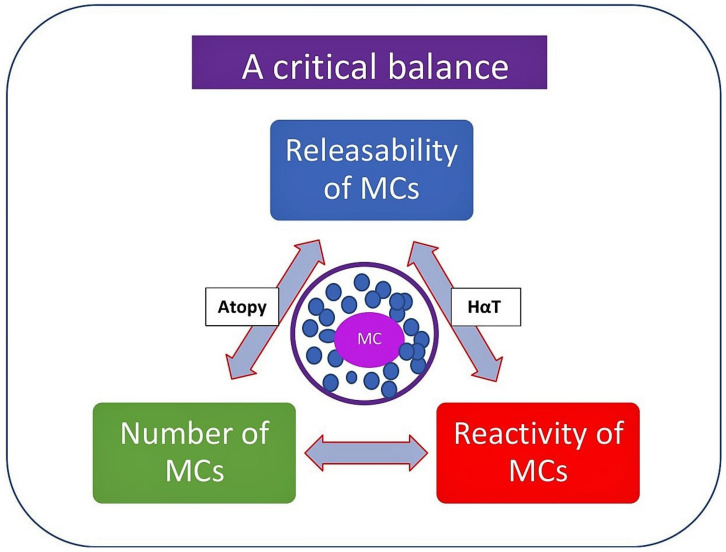
Most influential factors affecting the severity of symptoms of mast cell activation and mediator release. Please see the related text for further discussion regarding other potential factors determining the severity of symptoms. HαT, hereditary alpha-tryptasemia.

The symptoms of MC activation range from mild to severe to life-threatening and the severity of symptoms may depend on a number of factors (Figure 4). These include genetic predisposition; the number, reactivity, and releasability of MCs at the site; the organs affected; the type and route of allergen; and the type and consequences of comorbid conditions [54,55,56,57].

The release of MC mediators and the related symptoms show heterogeneity according to the tissue microenvironment in which MC activation occurs, as well as the trigger that causes the MC activation. Hence, the anatomic location of the MCs involved in the reaction and the magnitude of the mediators released determine the extent of clinical symptoms. The ability of MC to liberate mediators, also known as “releasability”, depends on several factors, including the underlying (primary) disease/pathology, the numbers and type of activated surface receptors, the signaling pathways involved, the type and route of allergen exposure, and the amount of allergen-specific IgE [12,54,55,56,57]. Moreover, coexisting genetic predispositions, such as the presence of atopy [58], PGE2 deficiency [59], or hereditary alpha-tryptasemia (HαT) [60,61,62], can also influence the severity of symptoms. Additional cofactors, like exercise, intake of NSAIDs or alcohol, and the presence of comorbidities (e.g., cardiovascular or respiratory), may also contribute to the development and severity of the symptoms of MC activation [54,55,56,63,64,65,66].

One of the most important factors influencing the severity of MC-activation-related clinical events is the presence of the underlying clonal MC disease. For instance, severe or even life-threatening MC activation events are an expected scenario in patients with mastocytosis due to the increased burden of MCs as well as the hyperreactive nature of MCs in these patients [12,19,30,67,68]. It is thought that a chronically activated KIT receptor in mastocytosis may also be responsible for the hyperreactive state of MCs. Indeed, in most subjects with SM, MCs exhibit the *KIT* D816V mutation (>90% of cases), and the KIT ligand stem cell factor augments IgE-dependent mediator release in normal MCs [12,64,69]. However, not all patients with SM bearing the *KIT* D816V mutation develop anaphylaxis. Therefore, additional genetic polymorphisms or mutations in the MC signaling components, other than the activating *KIT* D816V mutation, may contribute to excessive MC dysregulation and more susceptibility to anaphylaxis [12,64,69].

To explore the hyperreactivity issue further, a recent study was undertaken, and the authors surprisingly demonstrated that responsiveness to local activation of skin MCs using morphine and airway MCs using mannitol was similar in SM and healthy controls [29]. However, MCs cultured from mastocytosis patients showed higher reactivity with mannitol when activated in vitro [70]. Hence, there is as yet no substantial evidence that the MCs in mastocytosis are inherently hyperreactive in a way that lowers their threshold for activation or makes them more susceptible to massive degranulation. Nevertheless, this phenomenon remains to be the most likely explanation model.

In addition, the severity of anaphylaxis in mastocytosis may be also related to the extensive perivascular aggregation of MCs in these patients. Hence, the localization of MCs may be another important factor providing the rapid access of vasoactive mediators to the intravascular compartment, thereby predominantly causing hypotension during these reactions in mastocytosis [64,71].

### 4.1. Atopy

Atopy refers to a genetic predisposition to having an exaggerated immune response to allergens via the overproduction of IgE, and its inheritance pattern is believed to be polygenic [58]. Allergic asthma and allergic rhinitis are the most common manifestations of atopy followed by atopic dermatitis and food allergy. It is possible for two or more clinical diseases to coexist in an individual at the same time or at different times. Atopy affects a significant portion of the general population and often atopic individuals have a propensity to develop reactions to allergens. Thus, the prevalence of atopy ranges from 30% to 40% of the adult population in Westernized nations, and allergic asthma and allergic rhinitis are common conditions in the general population with a prevalence ranging from 10% to 30% [72,73,74].

Remarkably, however, the prevalence of atopy and atopic diseases is not found to increase in patients with MC disorders when compared to the general population [19,68,75]. In this regard, the prevalence of atopy was found to be 30% in SM patients based on allergen skin prick testing and/or the demonstration of specific IgE [19]. Likewise, the prevalence of an atopic disease was reported to be 22% in the same study [19]. Interestingly, when the authors further analyzed SM patients presenting with anaphylaxis and compared them to SM patients without anaphylaxis, significantly higher rates of atopy (42%) and atopic diseases (33%) in SM patients with anaphylaxis were found [19]. Thus, having atopic predisposition might be one of the modifying factors that may influence the prevalence and severity of symptoms related to MC activation, in particular in patients with SM.

### 4.2. Hereditary Alpha-Tryptasemia

Hereditary alpha-tryptasemia (HαT) is a recently identified autosomal dominant genetic trait that is characterized by excess copies of the alpha-tryptase gene, TPSAB1 [60,76]. The elevation in tryptase level is proportionate to the number of TPSAB1 genes and a median normal tryptase level is approximately 4.5 to 5 ng/mL. Nonetheless, in subjects with HαT, sBT levels are elevated and generally found to be higher than 8 ng/mL (often >10 ng/mL) [76]. Interestingly, they generally do not have increased urinary secretion of other MC mediators, such as prostaglandins or histamine metabolites.

HαT is the most common cause of elevated baseline tryptase, as it is found in approximately 6% of the general population [77]. However, the majority of individuals with HαT appear to be asymptomatic [78] and the prevalence of HαT among patients with common allergies is the same as that among unselected controls [77]. Nevertheless, the prevalence of HαT was reported to be increased in both severe venom-induced anaphylaxis and IA [61]. Furthermore, HαT is found to be two to three times more prevalent in patients with SM than in the general population [79], and additionally, patients with SM and concurrent HαT tend to present severe and more frequent symptoms of MC activation and anaphylaxis, in particular Hymenoptera venom anaphylaxis or IA [61,79,80]. Interestingly, however, the association between HαT and severe anaphylaxis appears to be independent of concomitant clonal mast cell disease [61]. One of the mechanisms involved in disease pathogenesis is the formation of α/β tryptase tetramers, which have been shown to cleave and activate EMR2 (EGF-like module-containing mucin-like hormone receptor-like 2) and PAR2 (protease-activated receptor 2) receptors [61]. The activation of these receptors may be involved in increased vascular leakage and edema formation, which subsequently leads to the manifestations of urticaria, angioedema, and anaphylaxis [61]. Although, at present, HαT alone is not considered an MC activation disorder, it is currently thought to be a modifying factor that may influence the prevalence and severity of anaphylaxis. Nevertheless, these findings need to be confirmed in larger prospective studies.

## 5. General Features of Anaphylaxis and MCAS in Mastocytosis

Adult patients with clonal MC diseases, i.e., mastocytosis and MMAS, have an increased susceptibility to severe anaphylaxis. This association has been clearly established and a number of studies reported a prevalence of anaphylaxis ranging from 22% to 49% in mastocytosis patients [19,68,75]. This represents an approximately 1000-fold increase in anaphylaxis as compared with the general population [81].

The triggers of anaphylaxis in adults with mastocytosis are various, including heat, cold, exercise, drugs, Hymenoptera venoms, and emotional stress, or may be unprovoked. Hymenoptera stings seem to be the most frequent elicitor [19,82] and a recent study reported a 28% overall prevalence in venom-induced anaphylaxis (VIA) among 122 patients with SM, which is clearly higher than in the general population [67]. Interestingly, VIA may be a presenting symptom that may lead to the diagnosis of SM. In this regard, one large study demonstrated that approximately 10% of 379 subjects with anaphylaxis due to Hymenoptera stings had elevated sBT levels (>11.4 ng/mL), and the majority of these subjects had SM or MMAS diagnosed after bone marrow biopsies [83].

In addition, food-induced reactions were also reported as an elicitor of anaphylaxis in SM [68,75]. However, most of those reactions remain patient-reported and unconfirmed. Therefore, interpretations of these data should be made with caution due to the lack of reliable confirmatory in vitro tests and the absence of provocation tests. Recently, a large, systematic study investigating food hypersensitivity and food-induced anaphylaxis (FIA) in mastocytosis subjects reported that the prevalence of FIA was at least 10-fold less compared with the prevalence of VIA in mastocytosis [84]. In rare cases, food-induced anaphylaxis may be the initial presenting symptom of mastocytosis. A case report presented a patient with more than 10 anaphylaxis episodes after eating meat, where a provocation test with pork resulted in delayed anaphylaxis, despite that the patient had only low levels of specific IgE to meats and galactose-alpha-1,3-galactose [85]. Further diagnostic workup confirmed underlying ISM [85]. Another report presented two patients with FIA, where fish was the trigger in both cases [86]. Furthermore, there are occasional case reports of patients with allergies to foods or preservatives [87]. Thus, cumulative clinical experience suggests that the incidence of IgE-mediated food allergy is not, or not fundamentally, increased in subjects with SM compared with that in the general population [84]. Moreover, some patients with SM complain about flushing and GI symptoms being triggered by histamine-rich diets, spicy foods, and alcohol; however, these symptoms rarely progress to anaphylaxis [84].

Likewise, data on patients with drug hypersensitivity and mastocytosis are scarce and the literature is largely limited to case reports [88]. Incriminating medications may include NSAIDs, antibiotics, angiotensin-converting enzyme inhibitors, general anesthetics, including opioids and muscle relaxants, and radiocontrast media [89,90]. Some patients with SM may be at risk for such reactions; however, the risk is probably lower in patients who previously tolerated such drugs and/or who have no history of anaphylaxis during anesthesia. At present, the available data in the literature are scant on this topic, and it is not possible to provide clear recommendations. Furthermore, unlike VIA, drug-induced anaphylaxis (DIA) is rarely associated with undetected MC disorder in the literature. In this regard, a study investigating patients with NSAID hypersensitivity and its correlation to occult SM failed to show elevated sBT levels [91]. Most recently, two large systematic studies reported varying degrees of NSAID-induced anaphylaxis among mastocytosis patients ranging from 3% to 9%, respectively [92,93]. In addition, a newly published study reported a low frequency of antibiotic-induced anaphylaxis in a large cohort of patients with mastocytosis, where the rate of anaphylaxis was only 0.8% [94]. Hence, it is presently inconclusive whether the incidence of DIA is increased in SM; however, the available data suggest that drugs are still uncommon elicitors when compared to VIA in mastocytosis.

Conversely, patients having IA, e.g., unprovoked anaphylaxis, is not a rare phenomenon in SM [19]. Indeed, there appears to be an intriguing relationship between IA and clonal MC disorders. Because unprovoked anaphylaxis may be the presenting symptoms of mastocytosis or MMAS, it is, therefore, essential to distinguish it from true IA [31,95,96]. Akin and colleagues reported the presence of a clonal MC population in 5 of 12 patients with IA in whom there were no features of UP [31]. Similarly, Gulen and colleagues performed bone marrow examinations in 30 cases of severe unprovoked anaphylaxis without signs of skin mastocytosis and demonstrated that 14 of these patients had clonally aberrant MC populations and were subsequently diagnosed with SM or MMAS [95]. Finally, more recently, 56 subjects with at least three annual episodes of unexplained anaphylaxis underwent bone marrow examination and the authors found evidence of clonal MC disease in 14% (8 of 56) [96]. The discrepancies among these studies may be due to the differences in referral patterns and in the diagnostic criteria for IA.

Another distinct feature of anaphylaxis in mastocytosis can be related to the clinical symptoms during anaphylaxis episodes. The most common clinical manifestations of anaphylaxis in these patients include severe cardiovascular signs and symptoms, such as hypotensive syncope [19,68,82], whereas urticaria, angioedema, and respiratory symptoms rarely occur [82]. Interestingly, studies with larger series from Europe reported that over 70% of anaphylaxis episodes among patients with SM presented with syncope/loss of conciseness irrespective of triggers [19,68,82]. These observations have led to essential clinical implications, as it has been challenging for clinicians to decide whether to pursue a further evaluation with a bone marrow examination in subjects presenting with anaphylaxis but no other features of SM, such as a lack of the typical skin lesions.

Consequently, certain screening tools have been developed over the years to predict underlying mastocytosis in patients presenting with episodes of severe anaphylaxis. The first such tool was developed by the Spanish Network for Mastocytosis (REMA) and they proposed that male gender, elevated sBT levels greater than 25 ng/mL, the presence of hypotension, and the absence of urticaria were all associated with a high risk for having underlying mastocytosis [82]. The REMA score showed a sensitivity of 92% and specificity of 81%, regardless of the trigger [82]. Subsequently, a modification of the “REMA score” has been introduced as the “Karolinska score,” using a reduced cut-off level of sBT (greater than 20 ng/mL, instead of >25 ng/mL) [95]. The modified version resulted in better sensitivity (93%) and specificity (94%) in patients with IA [95]. Most recently, a further variant of the previous tools has been proposed—the so-called National Institute of Health Idiopathic Clonal Anaphylaxis Score (NICAS)—using clinical symptoms, gender, and an sBT cutoff of 11.4 ng/mL and incorporating allele-specific polymerase chain reaction testing to detect the *KIT* D816V mutation in the peripheral blood [96]. Please see a detailed comparison of the different screening tools in Table 3.

Furthermore, other observational studies analyzed the potential risk factors for developing anaphylaxis in mastocytosis. It has been suggested that anaphylaxis occurs more often in patients with SM lacking mastocytosis in the skin [19,68] and in those with an atopic predisposition [19,67]. A male predominance has also been observed in patients with SM with anaphylaxis [75]. There are also data supporting an association between elevated sBT levels and an increased risk of severe anaphylaxis in indolent SM patients [68]. In other studies, however, severe anaphylaxis occurred preferentially in patients with a low burden of neoplastic MCs or did not correlate with higher sBT levels [19,67,97]. Moreover, the risk for anaphylaxis also appears to be significantly higher in patients with ISM as compared with the more advanced form of SM [19,68,75].

Recently, a systematic study was undertaken to determine predictive markers of developing de novo anaphylaxis in SM patients without a history of anaphylaxis [67]. After analyzing 122 patients with SM, with and without anaphylaxis, an anaphylaxis risk scoring tool to predict SM patients who had high risk versus low risk of developing anaphylaxis was proposed [67]. Accordingly, SM patients with anaphylaxis displayed unique clinical and laboratory features. Factors noted to significantly predict anaphylaxis in patients with SM were male gender, the absence of typical skin lesions of urticaria pigmentosa, an sBT level of less than 40 ng/mL, a serum total IgE level of more than 15 IU/mL, and the presence of an atopic predisposition [67]. The sensitivity and specificity of this tool were 86% and 54%, respectively. To this end, the correlation between higher sBT levels and the prevalence of anaphylaxis in patients with SM does not appear to be linear but shows rather a bell-shaped association where the risk is indeed lower with very high levels of sBT [67]. This finding is consistent with that of a study by van Anrooij et al. on VIA in patients with SM [97]. It should also be mentioned that although the total IgE levels are usually lower in patients with adult mastocytosis, SM patients who experienced anaphylaxis had significantly higher levels of total IgE [67]. Hence, these observations may support the existence of a distinct SM anaphylaxis phenotype.

Collectively, screening suspected patients for the diagnostic criteria and performing a comprehensive clinical workup, including detailed patient history, allergy tests and ultrasensitive molecular assays of *KIT* D816V, followed by applying the recommended diagnostic algorithms (e.g., REMA, Karolinska and/or NICAS), is crucial for diagnosing MCAS in patients with or without clonally aberrant MCs [98].

## 6. Management of Mast Cell Disorders

There is currently no cure for any variant of mast cell disorders; thus, the core component of the treatment is to control the symptoms caused by MC mediator release. Moreover, a stepwise, individual-based approach to the pharmacotherapy options appears to be a most convenient strategy for all patients [12,51,52]. Nevertheless, a detailed discussion of the management of these conditions is beyond the scope of this review and the reader is referred to excellent reviews on the topic in this issue of the journal.

Acute episodes of anaphylaxis and MCAS should be promptly treated with epinephrine, as evidence suggests that early treatment of systemic reactions prevents progression to more severe symptoms [99]. Intramuscular (IM) epinephrine is the drug of choice, as this drug reverses the inappropriate effects of the MC mediators produced during systemic activation [100,101]. Unfortunately, epinephrine is still underutilized, whereas corticosteroids are widely used as first-line therapy despite a lack of evidence [102]. In refractory cases of severe hypotension not responding to repeated doses of IM epinephrine, or hypotension followed by cardiac arrest, intravenous (IV) epinephrine should be given with continuous monitoring of cardiac response, blood pressure, and oxygen saturation [12,100,101]. Supplemental high-flow oxygen and IV fluid (e.g., normal saline) replacement should be administered. In addition, it is important to place the patient on their back, i.e., in the Trendelenburg position. With severe, unresponsive bronchospasm, an inhaled beta-agonist (e.g., salbutamol) can be given additionally. When the cardiovascular status and respiratory function stabilize, second-line medications such as H1-antihistamines (with or without H2-antihistamines) and corticosteroids are usually recommended [99]. However, the value of corticosteroids in the acute management of anaphylaxis is unclear because there is no substantial evidence to support their proposed effect on the prevention of protracted or biphasic reactions [102]. Regardless of the severity, all patients with such episodes should be kept under observation until their signs and symptoms have fully been resolved. The control of event-related tryptase levels may be beneficial when evaluating a patient’s reaction during the follow-up visit at an allergist/immunologist.

The principles of disease management of non-advanced variants of mastocytosis are focused primarily on trigger avoidance and symptom control; however, there are big variations among patients regarding the nature and sort of elicitors and the intensity and duration of the symptoms. Hence, prophylactic anti-mediator-type drugs or other pharmacological interventions can be individualized to specific needs and be prescribed for regular use or as required. For instance, a mastocytosis patient who is allergic to Hymenoptera venom and has experienced an episode of VIA should obtain lifelong venom immunotherapy [103]. Moreover, the symptoms of MCAS and mastocytosis can be managed by blockading the mediator receptors (e.g., H1 and H2 antihistamines or leukotriene receptor blockade), through the inhibition of mediator synthesis (aspirin/NSAID) or mediator release (sodium cromolyn), or using a combination of these approaches [12]. The use of other novel medications such as anti-IgE therapy (omalizumab) as an intervention has been recommended on a case-by-case basis [104,105]. Patients with mastocytosis, particularly those with advanced variants of the disease, may need a reduction in the number of MCs to prevent severe symptoms including anaphylaxis and/or progression to aggressive diseases [106,107,108,109,110,111,112,113].

## 7. Concluding Remarks and Future Directions

The nomenclature of disorders associated with pathologic MC activation is complex, as the same or similar conditions are described under a confusing variety of names. For instance, anaphylaxis, MCAS, and mastocytosis, although interrelated in terms of MC dysfunction, exhibit distinct clinical features and diagnostic approaches. Moreover, all three diagnoses may co-exist in one and the same patient; therefore, understanding the nuances of each disorder is crucial for healthcare professionals to provide accurate diagnoses and personalized management strategies. Hence, considering the complexity of these conditions, the care of such patients should optimally be provided in specialized centers or in close collaboration with experts of MC disorders. Furthermore, expanding knowledge on the presentation and triggers of anaphylaxis and MCAS among healthcare providers and the relatives of patients may increase patient safety by improving its recognition and management. Patients should also receive counselling on how to manage the reactions initially and when to seek emergency care help and should learn how to identify and avoid triggers in the first place.

Further research, through the investigation of intrinsic alterations in MCs causing cellular dysfunction, for instance, by utilizing proteomic-based approaches, including secretome analysis, may facilitate the development of novel biomarkers to distinguish patients with severe anaphylaxis and/or MCAS, as well as the design of new treatments targeting the intracellular signaling molecules of MC activation and mediator release [22,114,115,116]. Such approaches may provide important insight into patient outcomes and quality of life.

## Figures and Tables

**Table 2:** Clinical criteria of anaphylaxis (adapted from reference: [42]).

Anaphylaxis Is Highly Likely When Any One of the Following Three Criteria is Fulfilled		
**Criterion 1** Acute onset of an illness (minutes to several hours) with involvement of the skin, mucosal tissue, or both (e.g., generalized hives, itching, or flushing, swollen lips-tongue-uvula) AND at least one of the following: Respiratory compromise (e.g., dyspnea, wheeze-bronchospasm, stridor, reduced peak flow, hypoxia) Cardiovascular compromise (e.g., hypotension, syncope, collapse, incontinence)	**Criterion 2** Two or more of the following that occur rapidly after exposure to a likely *allergen* for that patient (minutes to several hours): Involvement of the skin or mucosal tissue Respiratory compromise Cardiovascular compromise Persistent gastrointestinal symptoms (e.g., crampy abdominal pain, vomiting, diarrhea)	Criterion 3 * Hypotension after exposure to known *allergen* for that patient (minutes to several hours)

* Low blood pressure (BP) is defined as systolic BP less than 90 mm Hg. or a decrease greater than 30% from that person’s baseline, or in Infants and children under 10 y: systolic BP less than 70 mm Hg + (2 × age in years).

**Table 3:** Comparison of different scoring tools, which are used in screening patients with anaphylaxis to evaluate the risk of underlying clonal mast cell disease (adapted from references: [82,95,96]).

	REMA Score		Karolinska Score		NICAS	
**VARIABLES**	Yes	No	Yes	No	Yes	No
**Male gender**	+1	−1	+1	−1	+1	−1
***Clinical symptoms***						
Angioedema	n/a	n/a	n/a	n/a	n/a	+1
Urticaria	n/a	n/a	n/a	n/a	+1	n/a
Flushing	n/a	n/a	n/a	n/a	−1	n/a
Urticaria/Pruritus/Angioedema	−2	+1	−2	+1	n/a	n/a
Syncope	+3	0	+3	0	+3	0
***Baseline tryptase***						
≤11.4 ng/mL	n/a	n/a	−1	n/a	−1	n/a
>11.4 ng/mL	n/a	n/a	n/a	n/a	+1	n/a
>20 ng/mL	n/a	n/a	+2	n/a	n/a	n/a
<15 ng/mL	−1	n/a	n/a	n/a	n/a	n/a
>25 ng/mL	+2	n/a	n/a	n/a	n/a	n/a
***KIT* D816V mutation**	n/a	n/a	n/a	n/a	+3	−1
Positive score *	≥2 points		≥2 points		≥2 points	
**Outcome**	High risk		High risk		High risk	

Abbreviations: REMA, the Spanish Network for Mastocytosis (REMA); NICAS, National Institute of Health Idiopathic Clonal Anaphylaxis Score; n/a, non-applicable; * The sum of positive and negative points of ≥ 2 is considered to be positive and indicates a high probability for underlying clonal mast cell disorders.

## Data Availability

Not applicable.
